# Efficacy and safety of CT-guided ^125^I seed implantation by coplanar template as a salvage therapy for vertebral metastases after failure of external beam radiation therapy: a retrospective study

**DOI:** 10.3389/fonc.2023.1084904

**Published:** 2023-04-28

**Authors:** Peishun Li, Yunling Bai, Qianqian Yuan, Qirong Man, Chao Xing, Yanchen Ren, Kaixian Zhang

**Affiliations:** Department of Oncology, Tengzhou Central People’s Hospital, Shandong, China

**Keywords:** CT-guided, 125I seed, coplanar template, vertebral metastases, external beam radiation therapy

## Abstract

**Purpose:**

To evaluate the efficacy safety of computed tomography (CT)-guided ^125^I seed implantation by coplanar template for vertebral metastases after failure of external beam radiation therapy (EBRT).

**Material and methods:**

Retrospective analysis of the clinical outcomes of 58 patients with vertebral metastases after failure of EBRT, who underwent ^125^I seed implantation as a salvage treatment with a CT-guided coplanar template-assisted technique from January 2015 to January 2017.

**Results:**

The mean post-operative NRS score decreased significantly at T_4w_ (3.5 ± 0.9, p<0.01), T_8w_ (2.1 ± 0.9, p<0.01), T_12w_ (1.5 ± 0.7, p< 0.01) and T_6m_ (1.2 ± 0.6, p< 0.01) respectively. The local control rates after 3, 6, 9 and 12 months were 100% (58/58), 93.1% (54/58), 87.9% (51/58), and 81% (47/58), respectively. The median overall survival time was 18.52months (95% CI, 16.24-20.8), and 1- and 2-year survival rates were 81% (47/58) and 34.5% (20/58), respectively. By performing a paired t-test analysis, there was no significant difference in D90, V90, D100, V100, V150, V200, GTV volume, CI, EI and HI between preoperative and postoperative (p>0.05).

**Conclusions:**

^125^I seed implantation can be used as a salvage treatment for patients with vertebral metastases after failure of EBRT.

## 1 Introduction

The vertebral column is the most common site of bone metastases (1, 2). More than 50% of patients with malignant tumors can develop or be diagnosed with spinal metastasis (3). Traditional treatments for vertebral metastases include surgery, radiation therapy, and chemotherapy. The efficacy of surgical treatment for spinal metastases is unsatisfactory and controversial (4). And it has certain limitations for the selection of surgical patients (5). Five to twelve percent of patients worsen neurologically after surgery (6–8). Radiation therapy plays an important role in the treatment of spinal metastases (9–11). But the spinal cord has a low threshold for radiation, making it impossible to increase the local dose of external radiotherapy, resulting in a low rate of local control of the tumor. It has been reported that more than one-third of patients with vertebral metastases who have received external radiation therapy have local recurrence (12).

With the development of minimally invasive therapy, radioactive seed implantation in the treatment of tumors has attracted more and more attention, and the scope of clinical applications has also been expanding.^125^I seed implantation has been widely used in the treatment of various malignant tumors, which has definite clinical efficacy (13–21). To our knowledge, there are few reports on CT-guided ^125^I seed implantation by coplanar template in the treatment for vertebral metastases.

This preliminary retrospective study was conducted to explore the efficacy and safety of ^125^I seed implantation under CT guidance by coplanar template for vertebral metastases after failure of external beam radiation therapy (EBRT). The research was approved by the Ethics Committee of Tengzhou Central People’s Hospital (approval no, 2021-Ethics Review-21).

## 2 Materials and methods

### 2.1 Selection of the patients

The inclusion criteria were: (1). Patients must have pathologically proven malignancy and radiographic evidence of vertebral metastases, number ≤3; (2). The pain of vertebral metastases was not relieved after previous therapy and pain score was not less than 4, Which was measured using a 0 to10 numeric rating scale (NRS) (22); (3). Except for vertebral metastases, no other organs had metastases or metastatic lesions were controlled;(4). No dysfunction of important organs, including heart, lung, kidney, etc.; (5). Karnofsky performance status (KPS) ≥70, and expected survival≥3 months; (6). All patients were discussed by a combination of radiation oncologists, medical oncologists, spine surgeons, pain medicine specialists, interventional radiologists, psychiatrists, and palliative care professionals before deciding on a course of treatment; (7). An informed consent for ^125^I seed implantation was signed by the patient or his legal guardian.

The exclusion criteria involved: (1). Patients with known Central Nervous System (CNS) metastases or a history of CNS metastases before treatment. For patients with clinically suspected CNS metastases, CT or MRI examination must be performed within 14 days before treatment to exclude CNS metastases; (2). Patients who had relapsed within 6 months after radiotherapy for vertebral metastases or had received radiotherapy within 6 months in adjacent vertebral sites; (3). Severe organ dysfunction;(4). Coagulation dysfunction, anticoagulant therapy should be stopped at least 5-7 days before implantation;(5). Poor general condition or cachexia;(6). No CT and other imaging data after ^125^I seed implantation.

### 2.2 Preoperative planning

Preoperative plan was delineated by clinicians, radiation oncologists and physicians together. CT scan was performed within 1 week before the treatment with a slice thickness of 5mm. The patient was placed in a prone position, secured by a vacuum negative pressure pad, with the centerline of setup marked on the body surface. CT images were transmitted to computer-assisted treatment planning-system to evaluate the feasibility of treatment and to design preoperative planning. Brachytherapy treatment planning system (BTPS, Beijing University of Aeronautics and Astronautics and Beijing Astro Technology Co. Ltd) was used. The prescription dose of this study was 120 Gy.

The radiation oncologist delineated the target volume and organs at risk (spinal cord, great blood vessels and adjacent tissues), set the prescribed dose and particle activity, determined the distribution and depth of the insertion needle, calculated the number of ^125^I seed and simulated the spatial distribution of particles. The 3D printing coplanar template (3DPCT) was made of corn resin and provided by Beijing Atomic Technology Co., Ltd, with the specifications of8 cm × 8 cm × 2 cm or 10 cm× 10 cm×2 cm. It was punched in accordance with 0.5cm spacing.

### 2.3 ^125^I seed implantation technique

For ^125^I seed implantation, a 64-row spiral CT scanner (Siemens, Germany) was used. The ^125^I seed was provided by Beijing Atomic Technology Co., Ltd (China), which was 0.8 mm × 4.5 mm (diameter × length) with a radioactivity of 0.4-0.8mCi and radioactive half-life of 59.6d. Supporting device for 3D PCT was connected to the bed. The puncture trajectory was marked on the skin. After skin disinfection and local anesthesia with 2% lidocaine, the coplanar template was placed on the patient skin entry point, which was consistent with the preoperative plan. All 18-gauge needles were inserted step-by-step into the lesions through the holes on the coplanar template. The material of the inner needle and the outer needle of the puncture needle were SUS304 cold-pressed steel plate specified by JIS G4305. When acquiring the CT half-way through needle placement, the needles may cause artifacts. It can be dealt with by adjusting the window width and window position of CT scan image appropriately. When all needles were deemed in place,^125^I seeds were implanted according to the preoperative plan. During the operation, radioactive particles should not be exposed in the air, to avoid causing unnecessary radiation to people around. After the operation, the instrument and the surrounding environment were detected by radiation monitors.

### 2.4 Postoperative dosimetry evaluation

**Figure 1:**
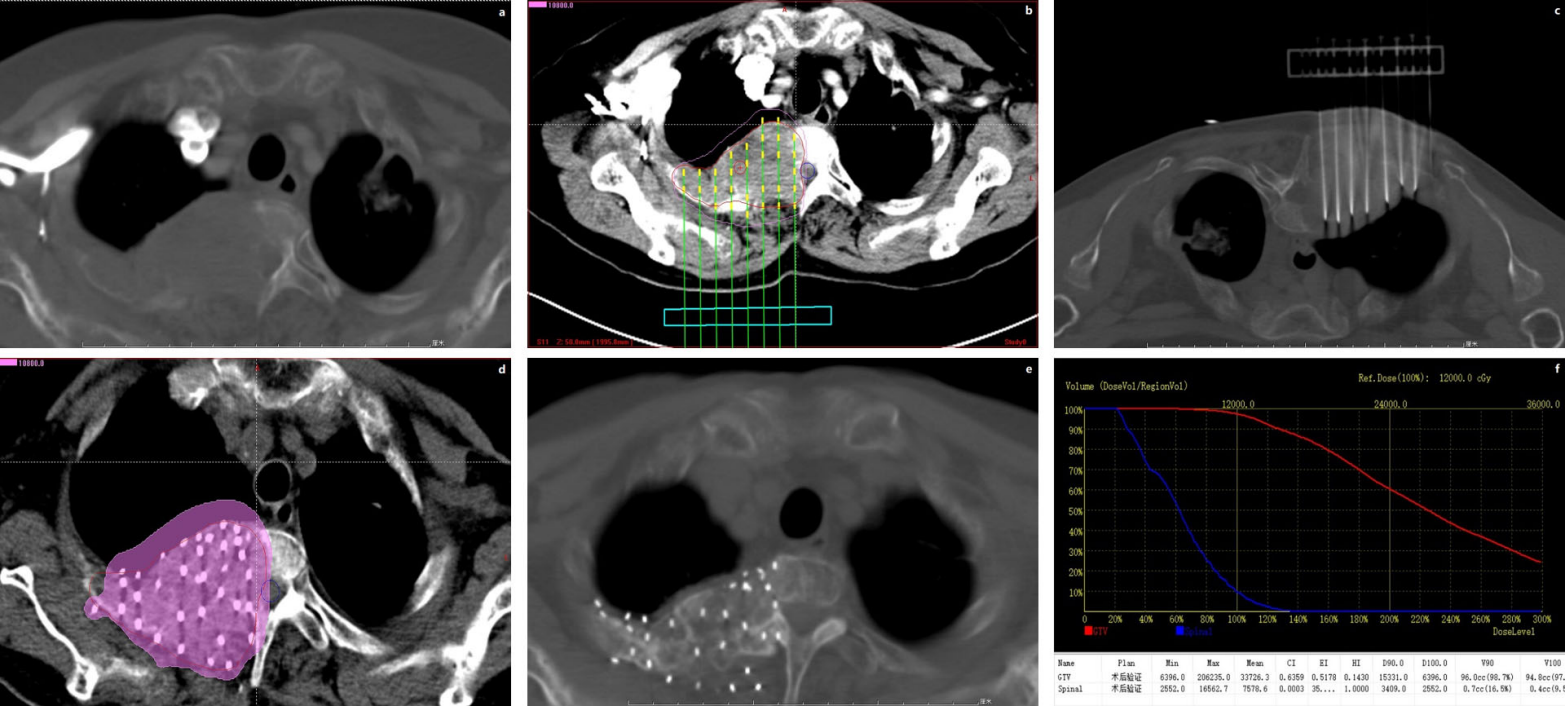
Represent CT scan of a patient during the whole treatment process. **(A)** Preoparative image. **(B)** Preoparative plan. **(C)** Operation process. **(D)** Postoperative Dosimetry Evaluation. **(E)** Twelve months after ^125^I seed implantation. **(F)** Postoperative Dose Volume Histogram.

CT scan was performed 3 days after operation to reduce the error of tumor volume due to tissue edema. And images were transmitted to TPS for dose verification (**Figure 1**). Dose parameters were calculated to evaluate the dose distribution, which include D90, D100, V90, V100, V150, V200, GTV volume, CI (23, 24) (Conformal index), EI (24) (External volume index) and HI (24) (Homogeneity Index).

CI is used to evaluate the degree of coverage to the target volume. CI = $\;\frac{V_{T,ref}}{V_{T}}\;\times \;\frac{V_{T,ref}}{V_{ref}}$ .

EI is used to evaluate overdosage to the surrounding tissues. EI = $\frac{V_{ref}\;V_{T,ref}}{V_{T}}$ .

HI is used to evaluate dose homogeneity within the target volume. HI = $\frac{V_{T,ref}\;V_{T,1.5ref}}{V_{T,ref}}$ .

$V_{T,ref}$ is volume of target receiving a dose equal to or greater than the reference dose; $V_{T}$ is volume of target; $V_{ref}$ is volume receiving a dose equal to or greater than the reference dose (treated volume); $V_{T,1.5ref}$ is volume of target receiving 150% of the reference dose.

### 2.5 Study end points

The primary outcome of our study was pain relief. NRS were used to evaluate the severity of patients’ pain, had been validated as an outcome measure (25–27). According to NRS for chronic pain, pain intensity at the treated vertebral level was evaluated and graded as follows:0, no pain; 1–3, mild pain; 4–6, moderate pain; and 7–10, severe pain. Patients completed NRS for the focal pain metastasis with the assistance of a trained visitor before surgery and 24 hours, 1 week, 4 weeks, 8 weeks, 12 weeks and 6 months after surgery.

Contrast-enhanced CT and MRI scans were performed 1 month after treatment and then every 3 months and compared with the post-procedural CT scans to identify local tumor progression. Local tumor progression was defined as an osteolytic defect of the tumor or growth of soft tissue components. Local control was defined as CR + PR + SD [LCR = (CR + PR + SD)/total], according to RECIST 1.1. Secondary outcomes were OS (time from the day of radioactive ^125^I seed implantation to death from any cause), preoperative and postoperative dosimetry evaluation.

Treatment-related adverse events were graded according to the National Cancer Institute Common Terminology Criteria for Adverse Events, version 3.0 and were coded and summarized according to the preferred terms in the Medical Dictionary for Regulatory Activities, version 15.0. American Spinal Injury Association (ASIA) International Standards for Classification of Spinal Cord Injury was used for neurological assessment (28).

### 2.6 Statistical analysis

SPSS 26.0 statistical software was used to analyze and compare all the data. The count data was analyzed by x^2^ test and expressed by [n (%)]. The measurement data was analyzed by t-test and expressed by ( $\bar{x}$ ± s). When p< 0.05, the difference has statistically significance. A two-sided test with P< 0.05 was considered statistically significant.

## 3 Results

### 3.1 General clinical information

58 patients (31males, 27females) were included in our analyses. The patients were aged from 28 to 82 years old, with an average age of (60 ± 13) years old. The primary tumor sites among the patients were lung cancer in26 (26/58, 44.9%), breast cancer in 15 (15/58, 25.9%), liver cancer in 7 (7/58, 12.1%), renal cancer in 4 (4/58, 6.9%), bladder cancer in 2(2/58, 3.4%), cervical cancer in 2(2/58, 3.4%), colon cancer in 1 (1/58, 1.7%), and esophageal cancer in 1 (1/58, 1.7%). A single lesion was treated in 45 (45/58, 77.6%) patients while two lesions were treated in 8 (8/58, 13.8%) patients and three lesions treated in 5 (5/58, 8.6%) patients, for a total of 76 lesions treated. The thoracic spine was the most common location for all vertebral metastatic lesions treated (36/76, 47.3%). There were 10 vertebral bodies with incomplete posterior margins (10/76, 13.2%). The patients’ characteristics were delineated in **Table 1**.

**Table 1:** Characteristics of patients before surgery.

Characteristics	Value
No. of patients (female/male)	27/31
Age, y ( ± SD)	60 ( ± 13)
Range	28-82
Mean Karnofsky performance status ( ± SD)	86 ( ± 7)
Previous treatment	
External beam radiation therapy	23/58 (39.7%)
EBRT and chemotherapy	35/58 (60.3%)
Opioid analgesics at presentation	50/58 (86.2%)
Primary tumor type histology (N=58)	
Lung Cancer	26 (44.9%)
Breast Cancer	15 (25.9%)
Liver Cancer	7 (12.1%)
Renal Cancer	4 (6.9%)
Bladder Cancer	2 (3.4%)
Cervical Cancer	2 (3.4%)
Colon Cancer	1 (1.7%)
Esophageal Cancer	1 (1.7%)
Vertebral metastasis’s location (N=76)	
Cervical Vertebra	5 (6.6%)
Thoracic Vertebra	36 (47.3%)
Lumbar Vertebra	28 (36.9%)
Sacrum	7( 9.2%)
Metastases numbers	
1	45/58 (77.6%)
2	8/58 (13.8%)
3	5/58 (8.6%)
NRS pain score	
01-Mar	0 (0)
04-Jun	38 (65.5%)
07-Oct	20 (34.5%)
Posterior margin of vertebral body	
complete	66 (86.8%)
incomplete	10 (13.2%)
Type of bone metastases (N=58)	
Osteolytic	35/58 (60.4%)
Osteoplastic	14/58 (24.1%)
Mixed	9/58 (15.5%)

EBRT, external beam radiation therapy; SD, standard deviation.

### 3.2 Pain relief

**Figure 2:**
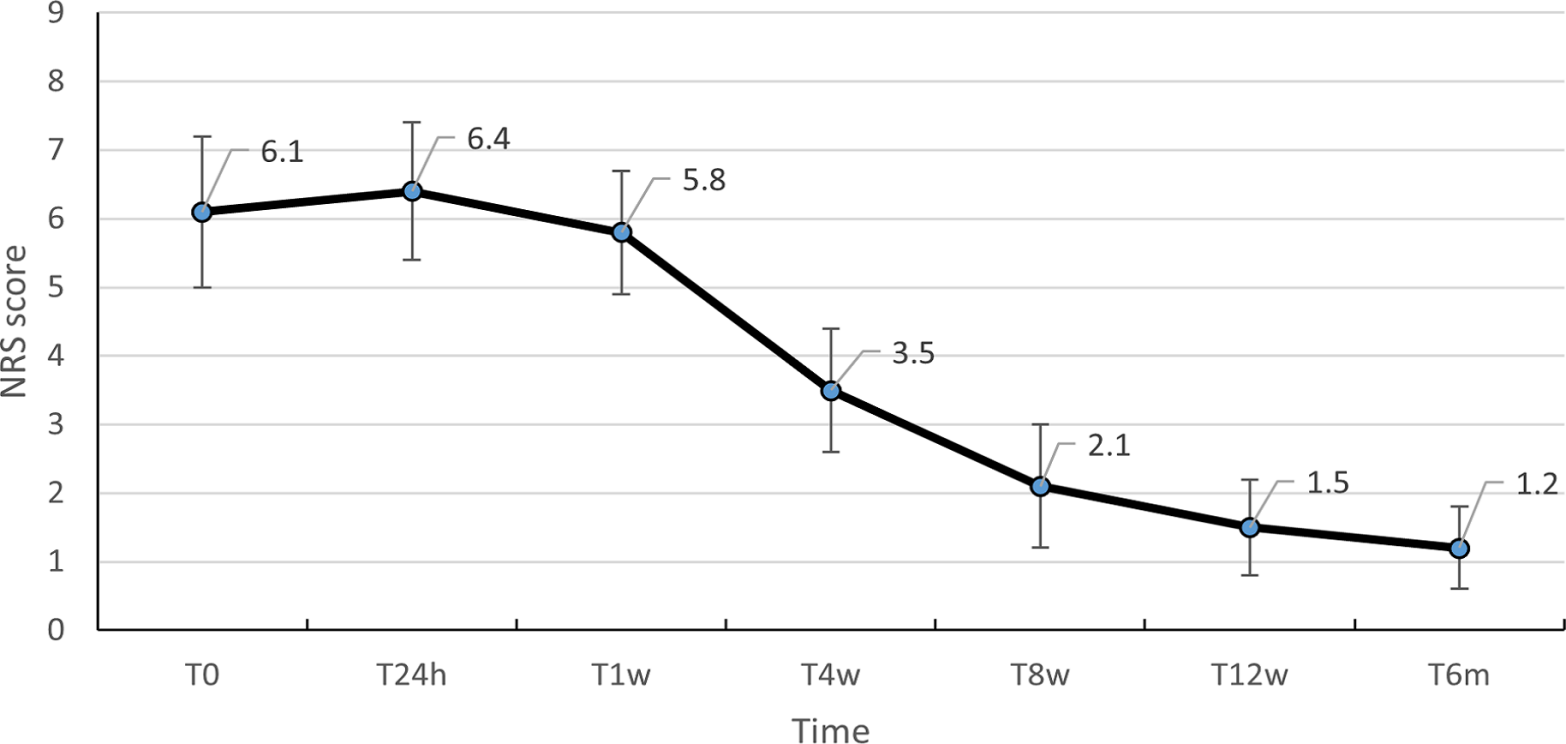
NRS score before and after the procedure. NRS, Visual Analog Scale. .

The NRS score for worst pain was 6.1 ± 1.1 before ^125^I seed implantation. The mean post-operative NRS scores decreased significantly at T_4w_ (3.5 ± 0.9, p<0.01), T_8w_ (2.1 ± 0.9, p<0.01), T_12w_ (1.5 ± 0.7, p< 0.01) and T_6m_ (1.2 ± 0.6, p< 0.01) respectively. There was no significant difference in scores among T_0_, T_24h_ (P=0.10) and T_1w_ (P=0.09) (**Table 2**, **Figure 2**).

**Table 2:** The NRS pain scores in each treatment period and distribution of pain severity scores.

	T_0_	T_24h_	T_1w_	T_4w_	T_8w_	T_12w_	T_6m_
	(n=58)	(n=58)	(n=58)	(n=58)	(n=58)	(n=58)	(n=53)
Average pain (0-10)							
Score ± SD	6.1 ± 1.1	6.4 ± 1.0	5.8 ± 0.9	3.5 ± 0.9	2.1 ± 0.9	1.5 ± 0.7	1.2 ± 0.6
P		0.10	0.09	<0.01	<0.01	<0.01	<0.01

24h, 24hours; 1w, 1week; 4w, 4weeks; 8w, 8weeks; 12w, 12weeks; 6m, 6months.

### 3.3 ^125^I seed implantation characteristics

All patients were successfully performed implantation at the first time. Median number of ^125^I seeds implanted was 55 (range, 10-96). The specific activity of seeds ranged from 0.5 to 0.8 mCi per seed, with a median of 0.7mCi/seed.

### 3.4 Local control and survival

No patients were lost to follow-up. The patients were evaluated radiographically for all of the ^125^I seed implantation procedures. The local control rates after 3, 6, 9 and 12 months were 100% (58/58), 93.1% (54/58), 87.9% (51/58), and 81% (47/58), respectively. The median overall survival time was 18.52months (95% CI, 16.24-20.8), and 1- and 2-year survival rates were 81% (47/58) and 34.5% (20/58), respectively.

### 3.5 Differences between pretreatment planning and postoperative dosimetry evaluation

There were 76 diseases in 58 patients. The dosimetric comparison before and after ^125^I seed implantation is shown in **Table 3**. There were no significant differences between preoperative and postoperative parameters, including D90,D100,V90,V100,V150,V200,GTV volume,CI,EI and HI, Which were compared by paired t-test (P > 0.05) (**Table 3**).

**Table 3:** Comparison of preoperative and postoperative dosimetry parameters of 76 lesions in 58 patients ( $\bar{x}$ ± s).

Parameters	Preoperative	Postoperative	P
D90 (Gy)	131.46 ± 20.15	135.13 ± 20.22	0.334
D100 (Gy)	75.92 ± 19.27	77.65 ± 19.26	0.634
V90 (%)	99.29 ± 1.01	99.40 ± 0.90	0.552
V100 (%)	98.43 ± 1.73	98.48 ± 1.53	0.866
V150 (%)	92.03 ± 7.54	90.94 ± 10.52	0.586
V200 (%)	82.89 ± 10.76	83.30 ± 10.56	0.841
GTV volume (cm^3^)	32.98 ± 22.63	33.72 ± 22.59	0.860
CI	0.40 ± 0.19	0.37 ± 0.19	0.456
EI	1.16 ± 0.60	1.23 ± 0.48	0.454
HI	0.12 ± 0.10	0.15 ± 0.14	0.216

D90, the dose delivered to 90% CTV; D100, the dose delivered to 100% CTV; V90, the volume to withstand 90% of the prescribed dose; V100, the volume to withstand 100% of the prescribed dose; V150, the volume to withstand 150% of the prescribed dose; V200, the volume to withstand 200% of the prescribed dose; GTV, Tumor target volume; CI, Conformal index; EI, External volume index of target area; HI, Homogeneity Index.

### 3.6 Side effects

All operations were completed successfully. Ten patients (17.2%, 10/58) appeared small volume subcutaneous hemorrhage, which may be associated with the injury of small subcutaneous vessels by puncture needle. All patients were relieved after pressing the puncture point to stop bleeding. Five patients (8.6%, 5/58) presented mild radiation dermatitis, but none developed radiation dermatitis above grade III. There were no deaths associated with ^125^I seed implantation. There were no serious complications such as infection, radiation osteonecrosis, or radiation myelitis during the follow-up period. Follow-up imaging showed that no patient had radioactive particle displacement.

## 4 Discussion

Spinal metastases are common in patients with advanced cancer, second only to lung and liver metastases in incidence (29). Pain is the most common complication in patients with bone metastases (30). Although EBRT has long been the main form of treatment for spinal metastases (9–11, 31), the effective rate of radiotherapy is about 60%, and about half of patients will experience a recurrence of pain within one year (32, 33).

Interstitial ^125^I seed implantation delivers a high local dose to tumors and sharply drops off at surrounding normal tissues (34). In recent years, with the wide application of ^125^I seed implantation in clinical practice, the relationship between dose and efficacy has gradually received attention, especially in prostate cancer (35). This study is the first to investigate the relationship between dosimetric factors and local control rate in the treatment of vertebral metastases with radioactive seed implantation. In this study, 58 patients were followed up for 4-35 months, with a median follow-up time of 17 months. The local control rates after 3, 6, 9 and 12 months were 100% (58/58), 93.1% (54/58), 87.9% (51/58), and 81% (47/58), respectively. The median overall survival time was 18.52months (95% CI, 16.24-20.8), and 1- and 2-year survival rates were 81% (47/58) and 34.5% (20/58), respectively. The NRS score for worst pain was 6.1 ± 1.1 before ^125^I seed implantation. The mean post-operative NRS scores decreased significantly at T_4w_ (3.5 ± 0.9, p<0.01), T_8w_ (2.1 ± 0.9, p<0.01), T_12w_ (1.5 ± 0.7, p< 0.01) and T_6m_ (1.2 ± 0.6, p< 0.01) respectively. The results show that ^125^I seed implantation has a good effect on the treatment of spinal metastases. In the cases of the present study, the mean NRS score of the patients at T_24h_ after surgery was slightly higher than the preoperative score, but the difference was not statistically significant.

At present, most doctors still use free-hand experience to implant radioactive seeds. This leads to inconsistent preoperative and postoperative doses, uncontrollable doses, and difficult to standardize technical means. In recent years, some studies (34, 36–38) have shown that 3DPCT assisted ^125^I seed implantation is a safe and effective method for the treatment of malignant tumors. However, there are few studies on coplanar template-assisted ^125^I seed implantation in the treatment of vertebral metastases. In this study, there were no significant differences in D90, D100, V90, V100, V150, V200, CI, EI and HI before and after implantation of 76 lesions, indicating the accuracy and consistency of this template.

The ideal method of CT-guided seed implantation should meet the following conditions: (1) It can effectively improve the accuracy of puncture; (2) It can visually display the position and puncture path of the puncture needle, and effectively guide physicians to avoid important organs; (3) It can shorten the operation time and reduce the occurrence of complications; (4) It can reduce the dose of X-ray radiation to patients and doctors.

CT-guided coplanar template assisted ^125^I seed implantation for malignant tumors is a new interdisciplinary technology, and the main advantages are as follows:(1) ^125^I seeds are low dose continuous irradiation;(2) It can ensure that the tumor target area gets a higher dose of irradiation;(3) Because the effective penetration distance of radioactive seeds is within 2cm, its penetration is limited, which can effectively protect the adjacent normal tissues;(4) Compared with the traditional single CT - guidance, this method may reduce the operation time.

Yang et al. (39) conducted a pig model experiment of percutaneous vertebroplasty combined with ^125^I seed implantation. None of the experimental pigs developed myelopathy, and pathological examination revealed no obvious cell damage. Our study showed that no serious complications occurred after ^125^I seed implantation, such as massive bleeding and radiation-induced myelitis. Therefore, we believe that ^125^I seed implantation may have a good safety in the treatment of spinal metastases.

To summarize the operation points of seed implantation in the treatment of vertebral metastases:

(1) The implanted seeds were arranged in a straight line in strict accordance with the Paris principles, so as to achieve parallel and equidistant implantation as far as possible. The CT scan can observe the tip position during implantation, which helps to make the particle distribution more accurate. Scan immediately after the operation to observe the position of seeds, supplement the distribution source if necessary, and rescan the lesions after satisfaction for postoperative verification and review.
(2) Patients with vertebral destruction and spinal cord compression should be treated with caution when puncture and needle insertion, especially those with obvious spinal cord compression. Preoperative MRI examination is recommended to clarify the relationship between the mass and the spinal cord, because sometimes the density of the mass and the spinal cord is similar on CT images, so that the boundary is unclear.
(3) For patients with metastatic vertebral bone destruction with intact cortical bone, due to the hardness of the cortical bone, 16G bone piercing needle can be used to penetrate the cortical bone, and then 18G particle piercing needle can be used to puncture the target under the guidance of CT. If necessary, an orthopedic hollow station will be used to assist puncture and punching. For patients with obvious vertebral destruction and partial cortical bone damage, 18G particle puncture needle can be directly used for puncture without bone puncture needle because of significantly reduced bone density.

Although this study reports encouraging results, several important limitations should be highlighted. Patients enrolled in this study undergoing ^125^I seed brachytherapy had multiple comorbidities or advanced systemic disease at baseline which may potentially confound treatment outcomes. In this study, the position taken by the patients in the imaging examination before particle implantation was not consistent with the position taken during particle implantation, which may lead to certain errors. If the patient’s position is consistent before and after surgery, the study may achieve better repeatability. This study showed a significant improvement in pain control and demonstrated a low complication rate, but most patients included studies had a short follow-up period. The local control rates were additionally challenging to summarize in our dataset due to the heterogeneous data across included studies. More prospective multicenter studies with a greater number of patients are needed to further demonstrate the effectiveness of this technique as a therapeutic option for spinal metastases after EBRT.

The results of this study showed that CT-guided coplanar template assisted with ^125^I seed implantation can effectively relieve pain in patients with vertebral metastases. In conclusion, CT-guided coplanar template assisted ^125^I seed implantation may be a viable salvage therapy in appropriately selected patients with painful vertebral metastases who were previously managed with conventional therapies.

## Data availability statement

The original contributions presented in the study are included in the article/supplementary material. Further inquiries can be directed to the corresponding author.

## Ethics statement

The studies involving human participants were reviewed and approved by the Ethics Committee of Tengzhou Central People’s Hospital (approval no, 2021-Ethics Review-21). The patients/participants provided their written informed consent to participate in this study.

## Author contributions

KZ conceived and designed the study. PL carried out the data collection, prepared the figures, and drafted the manuscript. YB and QY participated in the data collection. KZ, CX and QM performed seed implantation. YR carried out the dose calculation of seed implantation. All authors contributed to the article and approved the submitted version.
